# The mutation atlas of giant kelp (*Macrocystis pyrifera*): a mutation database resource for natural knockouts

**DOI:** 10.3389/fpls.2024.1338572

**Published:** 2025-01-27

**Authors:** Jose Francisco Diesel, Gary Molano, Sergey V. Nuzhdin

**Affiliations:** Department of Molecular and Computational Biology, University of Southern California, Los Angeles, CA, United States

**Keywords:** *Macrocystis pyrifera*, giant kelp, genetics, mutation, knockout, seedbank

## Abstract

Giant kelp (*Macrocystis pyrifera*) is a paramount species of immense ecological and economic importance. It forms dense underwater forests, providing crucial habitat and serving as a foundation species for diverse marine ecosystems. Understanding the genetics of giant kelp is essential for conservation and sustainable farming, safeguarding these valuable ecosystems and their benefits. By analyzing mutations based on their impact, we can gain insights into the potential functional consequences and implications for the organism, helping to identify critical genes or regions that may play a significant role in adaptation, development, and environmental response. To achieve this, we annotated the effects and impact of spontaneous mutations in 559 giant kelp individuals from four different populations. We found over 15.9 million mutations in genes of giant kelp, and classified them into modifier, low, moderate, and high impact depending on their predicted effects. The creation of this mutation effect database, attached to the seedbank of these individuals, offers several applications, including enhancing breeding programs, aiding genetic engineering with naturally occurring mutations, and developing strategies to mitigate the impact of environmental changes.

## Introduction

Giant kelp (*Macrocystis pyrifera*) is a prominent marine organism that plays a significant role in maintaining the ecological balance of coastal ecosystems (Graham et al., 2007; Reed and Brzezinski, 2009). As the largest and fastest-growing species of algae on Earth, it provides habitat, food, and shelter for a diverse range of marine species (Edwards et al., 2015). However, giant kelp populations are facing unprecedented challenges due to increasingly frequent and severe heatwaves. These heatwaves have negatively impacted giant kelp, leading to episodes of mass die-offs and threatening its genetic diversity (Filbee-Dexter et al., 2020; Smale, 2020). Beyond its ecological significance, giant kelp holds immense promise for human benefit, particularly in the fields of kelp farming and ecosystem restoration (Campbell et al., 2014; Buschmann et al., 2017; Layton et al., 2020).

Giant kelp farming has garnered growing interest as an alternative means of bolstering the world’s food supply, producing biofuels, and sequestering carbon dioxide from the atmosphere (Duarte et al., 2021; Spillias et al., 2023). The cultivation of giant kelp has demonstrated remarkable potential for sustainable aquaculture practices due to its rapid growth rates, ability to absorb excess nutrients from surrounding waters, and its lack of competition for agricultural land, fertilizer, or freshwater resources (Gerard, 1982; Stewart et al., 2009; Rassweiler et al., 2018). Recent advances in kelp genetics, including the release of many high quality annotated genomes, are helping to unlock the vast potential of genomics in improving giant kelp commercial cultivation and restoration efforts (Paul et al., 2022; Denoeud et al., 2024).

Genetic resources, including high-quality annotated reference genomes and a sequenced founding populations, are essential for advancing breeding programs and maximizing desirable traits in crops (Galluzzi et al., 2020; Pour-Aboughadareh et al., 2021). By utilizing comprehensive genetic data, scientists and farmers can identify and select individual kelp specimens with favorable traits, such as enhanced growth rates, improved nutritional content, and heightened resistance to environmental stressors (Briggs, 1998). Genetic information could aid in developing novel techniques to cultivate kelp strains better adapted to diverse oceanic conditions and various aquaculture systems. Moreover, studying naturally occurring mutations provides an alternative to artificially produced mutations and offers a valuable approach to addressing some concerns associated with genetically modified organisms, with benefits in permitting, safety perception, and preserving genetic diversity. Over 60 newly annotated brown macroalgae reference genomes, including two giant kelp genomes, greatly increase the genetic resources available to aid in giant kelp breeding. However, the lack of whole-genome mutational data from many individuals across different giant kelp populations hinders genomics-assisted breeding efforts (Paul et al., 2022; Diesel et al., 2023; Denoeud et al., 2024).

This research contributes to genomic tools bridging the macroalgae knowledge gap by providing a detailed study of the mutation landscape of giant kelp. It aims to uncover insights into mutation patterns, selective pressures, and create a mutation effect database for a seedbank, thereby facilitating breeding efforts and scientific research. By understanding the genetic basis of desirable traits and naturally occurring mutations, this research aims to pave the way for the sustainable and efficient cultivation of giant kelp while contributing to the preservation of marine biodiversity in the face of environmental challenges.

## Results and discussion

**Figure 1:**
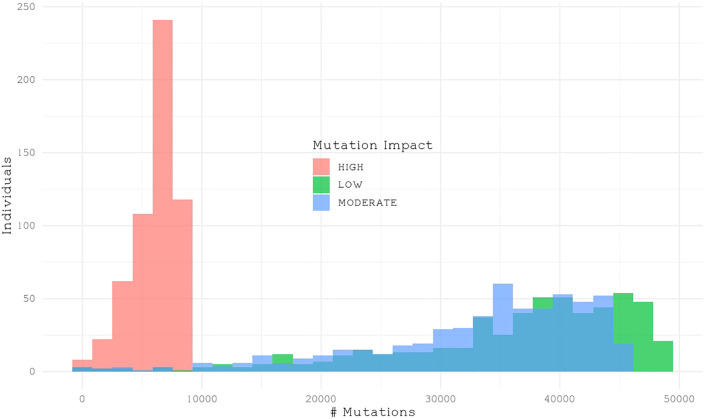
Histogram showing the number of impactful mutations per individual.

**Figure 2:**
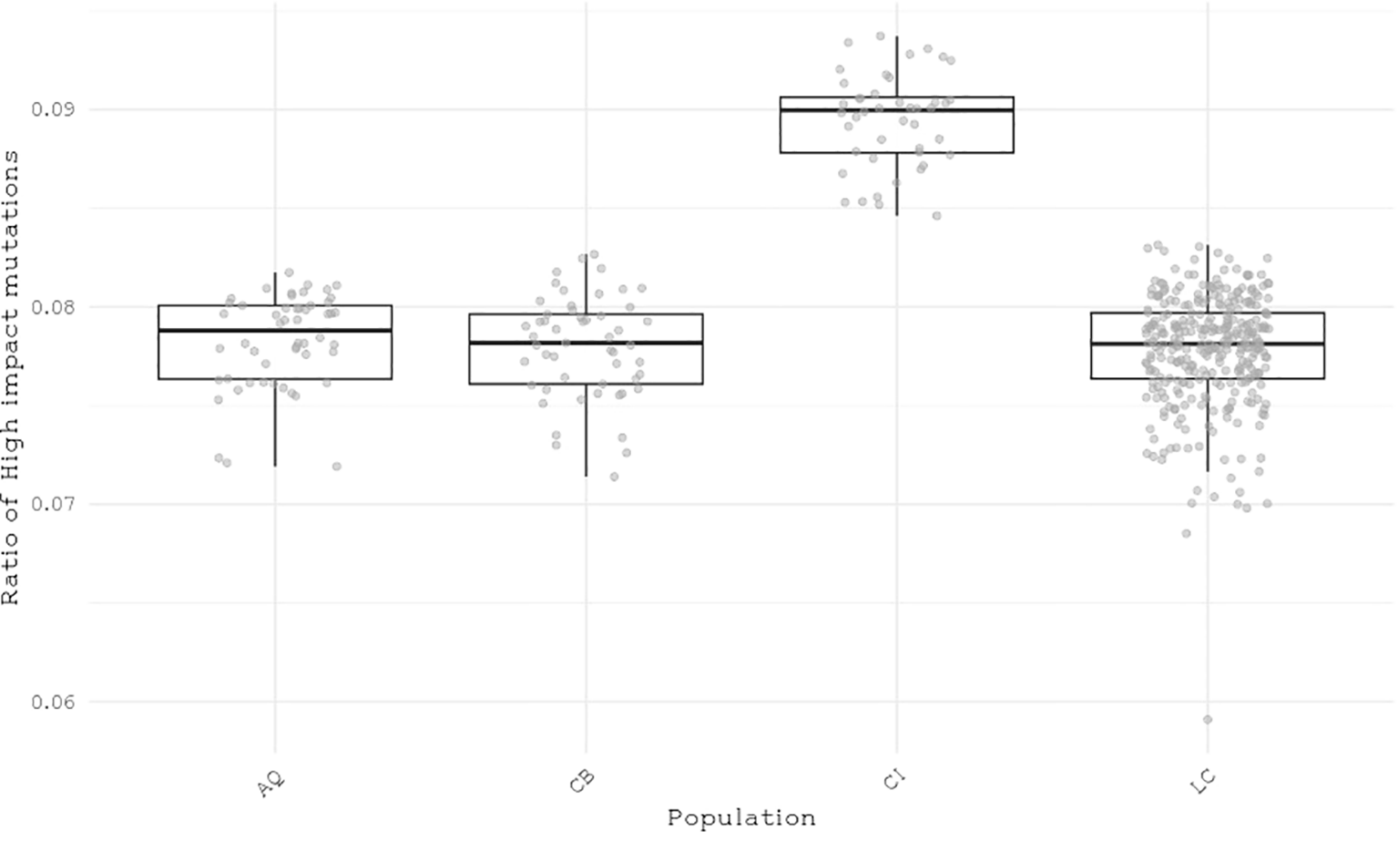
Distribution of the ratio of high-impact mutations per individual to the total number of impactful mutations across four populations.

We classified single nucleotide polymorphisms (SNPs) from 559 individuals from four populations (Catalina Island [CI], Camp Pendleton [CP], Arroyo Quemado [AQ], and Leo Carrillo [LC]) into modifier, low-impact, moderate-impact, and high-impact mutations based on their predicted effects on gene sequences, according to SnpEff list of effects (Cingolani et al., 2012). All four populations are located within a narrow band between 11 m and 1.8 km from the shoreline of Southern California, with yearly average sea surface temperatures ranging from 15.5°C to 17.7°C (**Table 1**), and have previously been identified as distinct populations (Johansson et al., 2015). We found over 28 million variants, resulting in more than 42 million predicted effects. Of those, 97% were modifiers, expected to have no significant impact on a gene. Low- and moderate-impact mutations account for 1.5% and 1.4% of mutations, respectively, while high-impact mutations make up 0.05% of all mutations (**Table 2**). The average number of low-, moderate-, and high-impact mutations per individual is approximately 36,100, 33,300, and 6,000, respectively (**Figure 1**). The Catalina Island population exhibited a disproportionately higher ratio of high-impact mutations compared to the other populations (**Figure 2**). Further investigation into the underlying causes of this disparity could provide valuable insights into the genetic adaptations of giant kelp in response to local conditions.

**Table 1:** Sampling location for all populations, average sea surface temperature (SST) in degrees Celsius, and distance from shore in meters.

Population	Average SST (°C)	Distance from shore (m)	Latitude	Longitude
Arroyo Quemado	15.59	198	34.468783	− 120.121417
Catalina Island	17.73	11	33.446747	− 118.485044
Camp Pendleton	18.05	1,880	33.290910	− 117.490997
Leo Carrillo	17.11	67	34.042933	− 118.934500

**Table 2:** Predicted impact of mutations, including their respective counts and the percentages they represent within the total mutation dataset.

Impact	Count	Percent
Neutral	41,710,434	97.793
Low	437,670	1.026
Moderate	456,837	1.071
High	46,825	0.11

**Figure 3:**
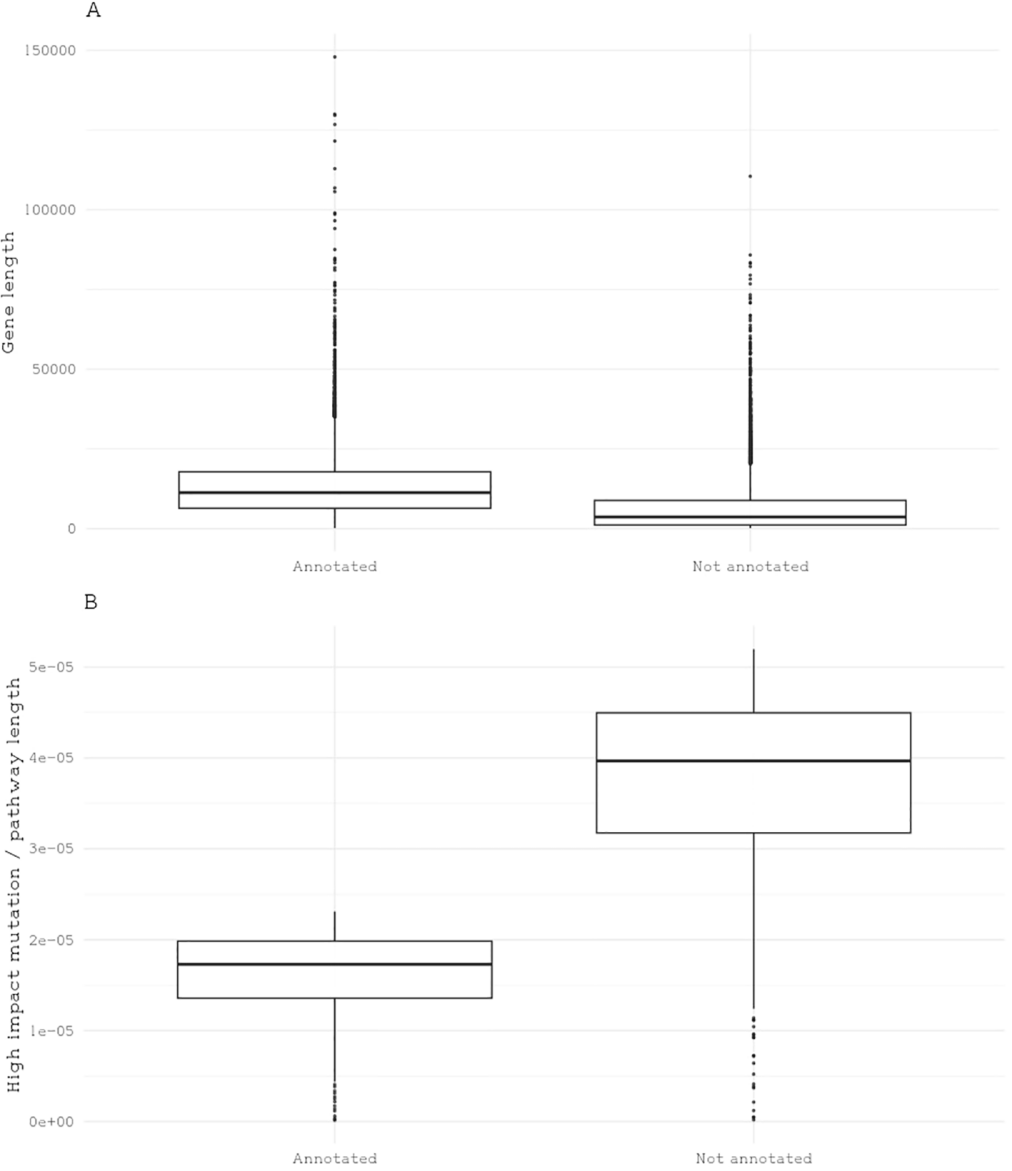
Representation of gene length and the quantity of high-impact mutations based on annotation status. **(A)** Boxplot representation of gene length distribution categorized by gene annotation status. **(B)** Boxplot depiction of the number of high-impact mutations in annotated and nonannotated genes, with each dot representing a gene.

Genome annotation often relies on comparative genomics, where similarities between the target species’ genes and those of well-annotated model species are used to infer gene function. In the case of brown macroalgae like giant kelp, recent advances in kelp genomics increase the resources available for comparative studies (Cormier et al., 2017; Shan et al., 2020; Paul et al., 2022; Denoeud et al., 2024). However, the novel pathways found in brown algae, such as alginate metabolism, require additional validation (Mazéas et al., 2024). While increasing sequencing data for kelp is important, integrating experimental approaches—such as gene knockdowns, overexpression studies, or associating specific genes with phenotypic traits—will be key to advancing our understanding of kelp’s gene functions. The giant kelp genome contains 25,900 genes, but only about 8,000 have known annotated functions and are mapped to Eukaryotic Orthologous Groups (KOG). Here, annotated genes tend to be larger in size and have fewer impactful mutations compared to genes that are not annotated (**Figure 3**). This suggests a higher degree of conservation among annotated genes, likely due to factors such as ascertainment bias in annotation and the unique functional specialization of giant kelp, which diverges from traditional model organisms (Armengaud et al., 2014; Parikesit et al., 2014).

### Mutation distribution and selective pressure

**Figure 4:**
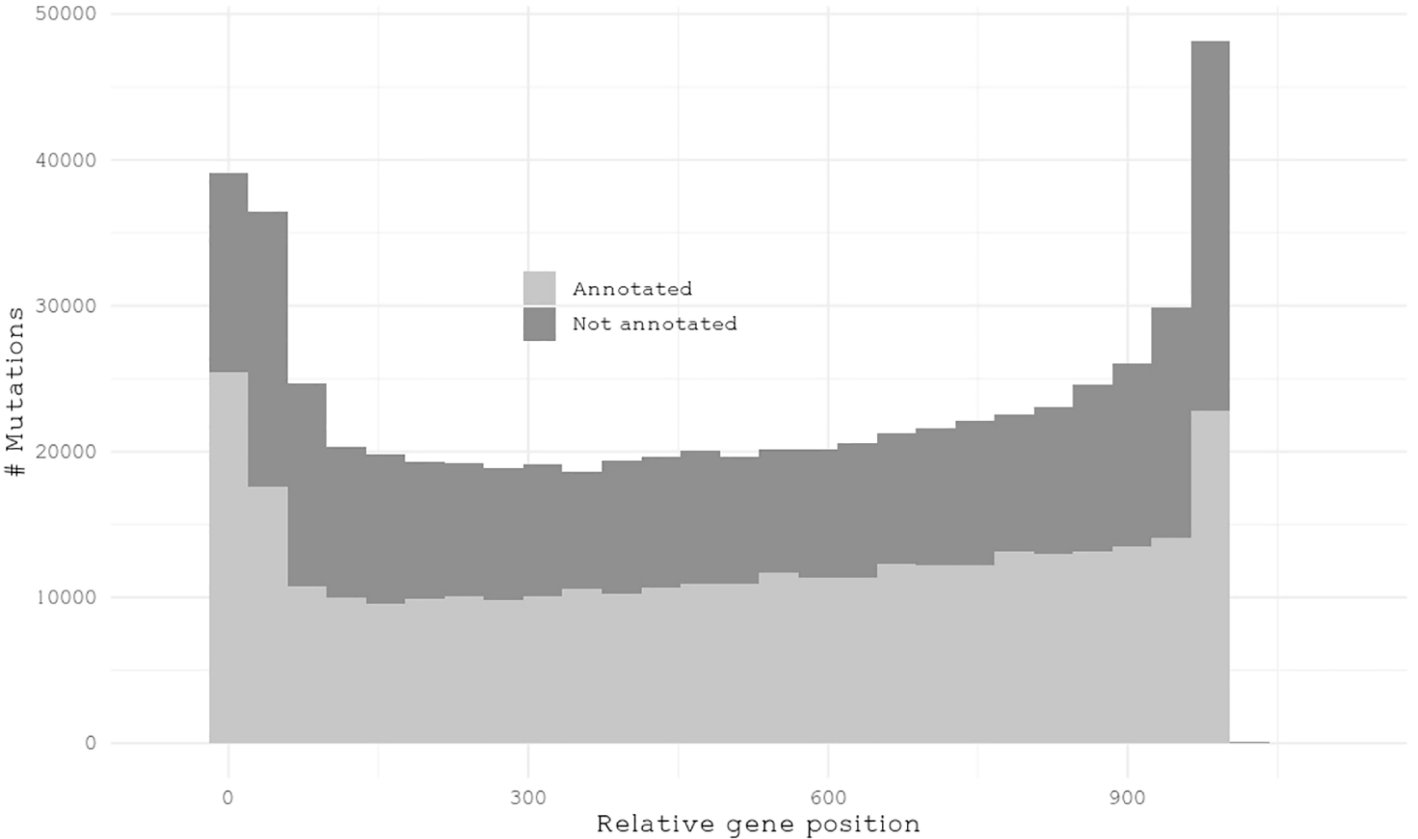
Histogram illustrating the frequency distribution of mutations normalized to a relative gene length of 1,000 nucleotides.

**Figure 5:**
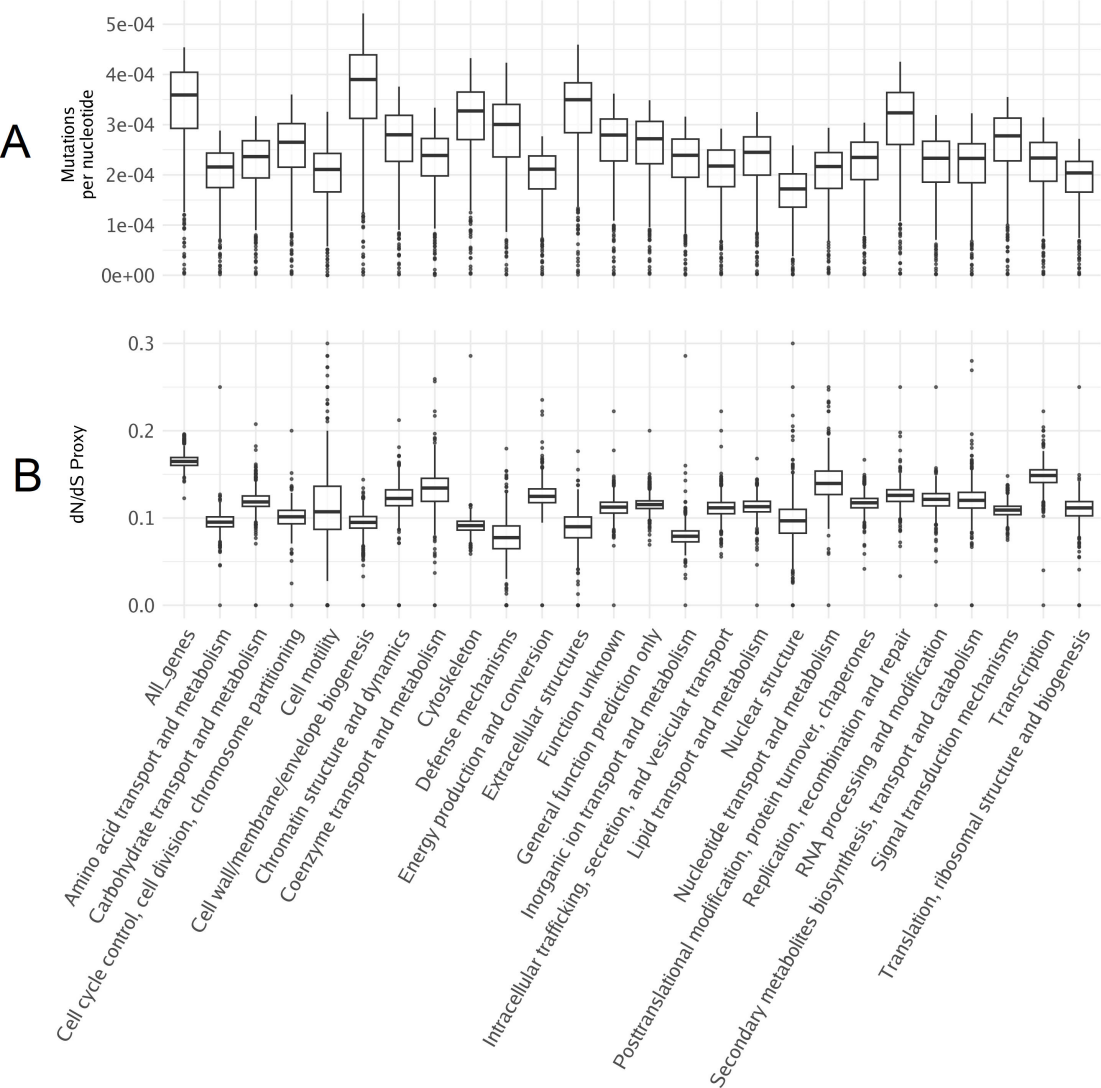
Mutation distribution and selective pressure. **(A)** The sum of all effect mutations in a pathway normalized by the total pathway length per individual. **(B)** Proxy for dN/dS, where high- and low-impact mutations serve as nonsynonymous and synonymous mutations, respectively. Each point represents an individual.

While we will focus on annotated genes in giant kelp, we first examined the mutation distribution across all genes. Regardless of their number, mutations are distributed similarly between annotated and unannotated genes in terms of their relative position within a gene (**Figure 4**). To understand how mutations were distributed among annotated KOG genes, we calculated the sum of all effect mutations relative to the total length of the annotated genes in each pathway per individual. This approach provides insights into the mutational landscape across different biological pathways. Pathways exhibiting the smallest number of mutations relative to their size were *nuclear structure* and *translation*, *ribosomal structure*, and *biogenesis*, with means of 1.6 × 10^−4^ and 1.9 × 10^−4^, respectively. These pathways appear to be relatively conserved, likely due to selective pressure, indicating strong evolutionary constraints acting upon them. The pathways with the highest number of mutations relative to pathways size were *extracellular structures* and *cell walls*, with means of 3.3 × 10^−4^ and 3.6 × 10^−4^, respectively (**Figure 5**). These findings underscore how the pathways involved in brown algal cell walls and extracellular components may accumulate mutations to help them interact with the dynamic marine environment, including responses to climate stressors such as warmer and more acidic water, as wells as pathogen defense (Michel et al., 2010; Wernberg et al., 2018; Wu et al., 2019).

We can use nonsynonymous substitutions (dN)/synonymous substitutions (dS) to measure whether natural selection is acting to preserve or change amino acid sequences in a protein (Miyata and Yasunaga, 1980). dN/dS is derived from comparing the rates of nucleotide substitutions that result in changes to the amino acid sequence of a protein (dN) with the rates of nucleotide substitutions that do not change the amino acid sequence (dS) (Johansson et al., 2015). Here, we used high- and low-impact mutations as proxies for non-synonymous and synonymous substitutions, respectively, to identify which pathways are the most constrained by selection. The pathways with the lowest ratio of high/low mutations were *defense mechanisms* and *inorganic ion transport and metabolism*, while *transcription* and *nucleotide transport and metabolism* exhibited the highest ratio (**Figure 5**). It is worth mentioning that genes in annotated pathways are more constrained when compared to all genes in the genome, further highlighthing the bias toward conserved genes in annotations.

### Population genetics and selection

**Figure 6:**
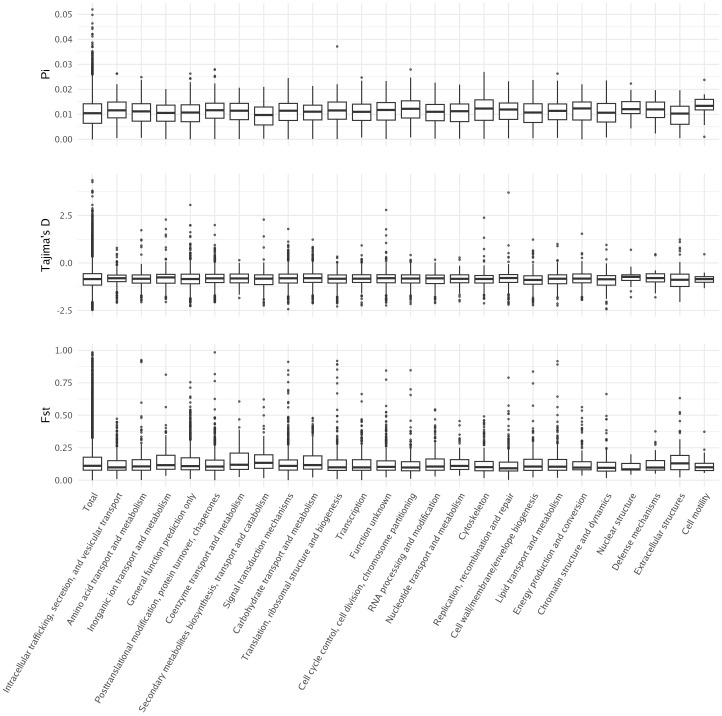
Distribution of Tajima’s D, nucleotide diversity, and Fst across genes annotated with KOG categories.

To better understand whether any set of genes was under selection, we first calculated nucleotide diversity (Pi), Tajima’s D, and Fst for each gene in the giant kelp genome (**Figure 6**). The average nucleotide diversity in giant kelp genes was 0.0105 (Nei and Li, 1979). Previous measurements of nucleotide diversity in giant kelp, conducted by Molano et al., were based on a set of highly conserved genes and showed an average nucleotide diversity five times smaller (Molano et al., 2022). Our whole-genome results indicate a moderate level of genetic variation within giant kelp, suggesting that the genome harbors considerable genetic diversity, which may support adaptation and evolutionary resilience in changing environments.

Tajima’s D was slightly negative at − 0.839, indicating an excess of low-frequency mutations (Tajima, 1989). Similar values of Tajima’s D in giant kelp were found in previous studies, suggesting purifying selection (Molano et al., 2022). The average Fst was 0.146, which is relatively high and reflects a significant degree of differentiation among the three populations studied, consistent with previous studies (Johansson et al., 2015; Molano et al., 2022; Gonzalez et al., 2023). No significant difference was found between different gene functions when using these population genetics metrics. This suggests that selective pressures acting on specific gene functions are not readily apparent from these metrics alone, or the limited number of annotated genes may not be large enough to obtain a signal. Therefore, further investigations are needed to uncover potential adaptive patterns.

### Biomass

**Figure 7:**
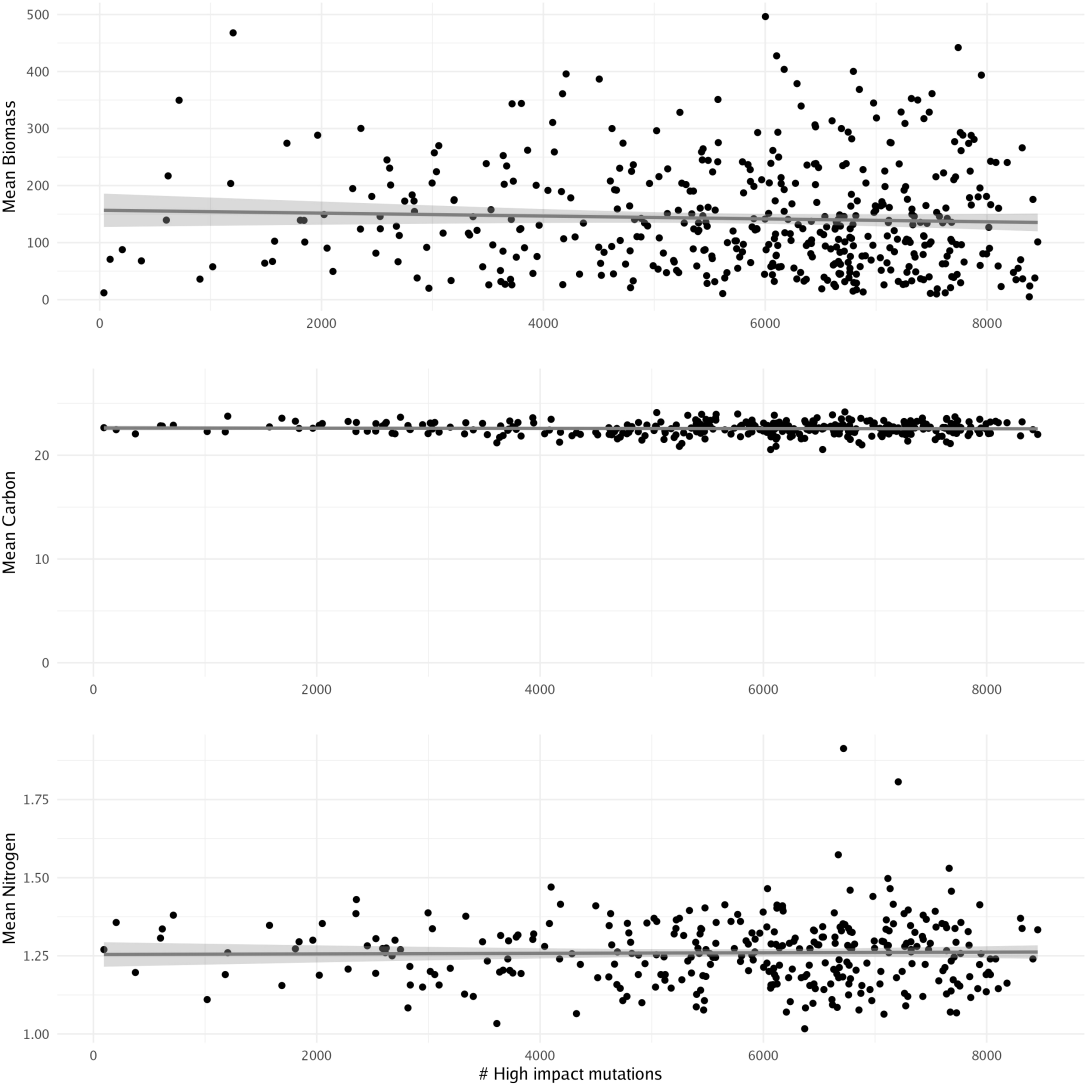
Linear model showing the relationship between the number of high-impact mutations and mean biomass, carbon, and nitrogen concentrations. No significance was found using a linear model.

Predicting biomass in kelp farming can significantly influence breeding programs by providing valuable data and insights that enhance the efficiency and success of selective breeding initiatives. These breeding programs aim to develop kelp varieties with desirable traits such as fast growth, high biomass production, disease resistance, and improved product quality. Previously, 500 females from the four populations were crossed with one male from Leo Carillo and outplanted at a kelp farm near Santa Barbara, CA. Biomass, carbon, and nitrogen data were then collected from the resultant sporophytes (Miyata and Yasunaga, 1980). In this context, we aimed to explore the potential influence of high-impact mutations on an individual’s biomass, carbon, and nitrogen levels. We assessed the correlation between these factors and the number of high-impact mutations. However, we found no significant association between any of the factors and the number of high-impact mutations when using a linear model (**Figure 7**). This suggests that the number of high-impact mutations may not directly influence biomass differences in giant kelp, indicating that more comprehensive modeling approaches, such as genome-wide association studies, may be needed for more accurate biomass predictions and to identify other economically relevant traits.

### Resource for natural knockouts

The discovery and documentation of existing knockouts in wild giant kelp populations offer a compelling advantage when seeking permits for farming and restoration efforts. Unlike introduced genetic modifications, these knockouts result from spontaneous genetic mutations that occur without targeted intervention, maintaining the existing genetic diversity within the species (Monroe et al., 2020). Consequently, using kelps with spontaneous knockouts may help alleviate some of the concerns and regulatory hurdles associated with genetically modified organisms.

**Figure 8:**
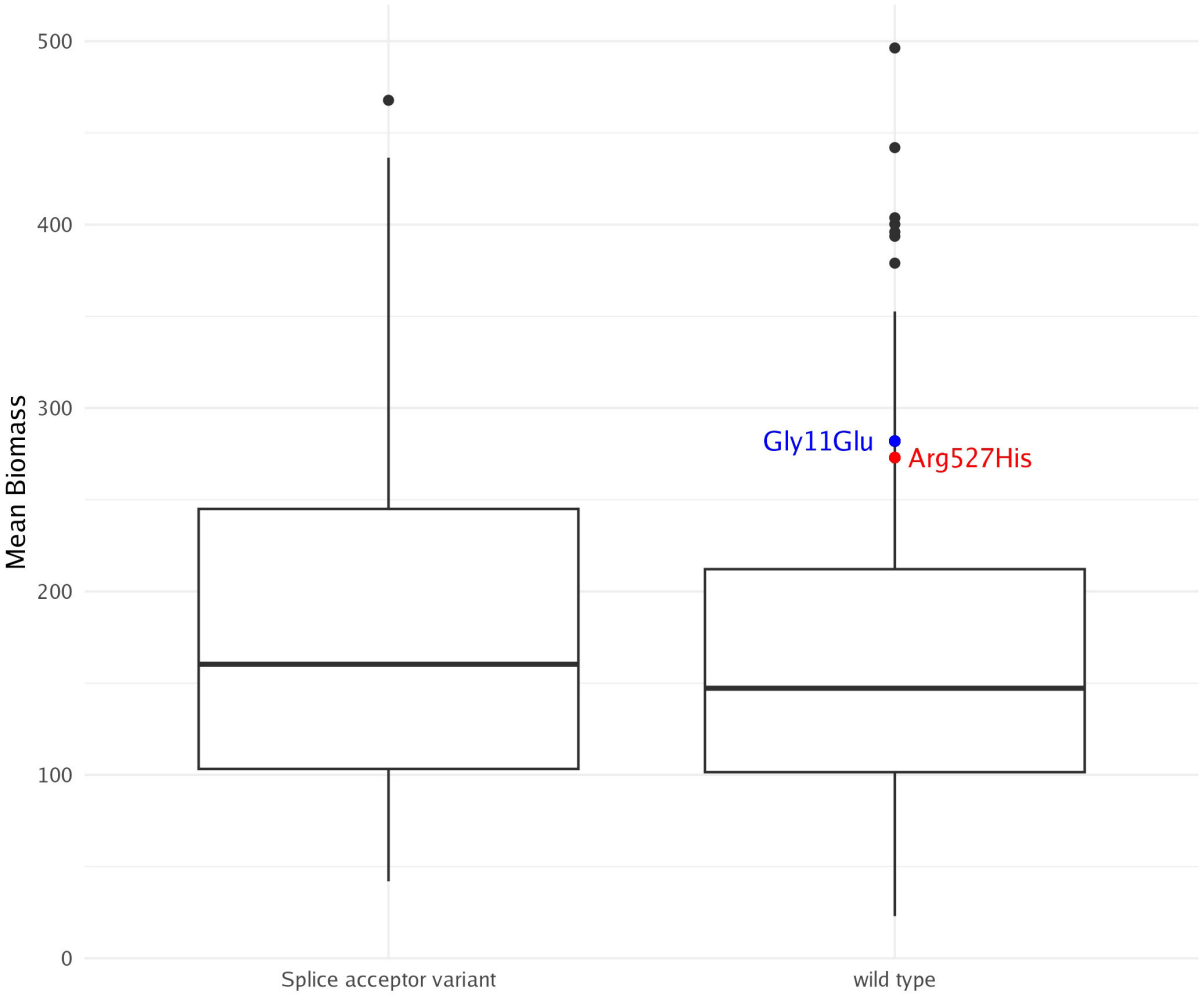
AMP deaminase mutations and their possible effect on biomass. No significant differences were observed between the splice acceptor variant and wild-type variant. Blue and red dots represent individuals with respective missense mutations, neither of which carry the high-impact splice acceptor variant.

Knockouts and knockdowns have emerged as powerful tools in algae research, significantly enhancing photosynthetic productivity, biomass yield, and cold tolerance (Baek et al., 2016; Kotchoni et al., 2016). A well-documented example is the knockdown of AMP deaminase, leading to remarkable improvements in biomass production, cold tolerance, and oil content in green algae (Kotchoni et al., 2016). As a testament to the database’s utility, we explored AMP deaminase mutations. Among the variants, three were categorized as moderate-impact missense mutations, while one was identified as a high-impact splice acceptor variant. Two individuals harboring distinct missense mutations (Gly11Glu and Arg527His) exhibited higher biomass than the average of the entire sample. However, the third missense mutation, present in seven individuals, showed no observable impact on biomass. The high-impact splice acceptor variant was found in 70 individuals within the cohort. While this variant appears to be associated with a slight increase in biomass among carriers, statistical analysis did not reveal significance (**Figure 8**). CRISPR/Cas-9 offers a highly efficient and targeted method for modifying genes, enabling precise alterations that can enhance traits like photosynthesis, growth, and stress tolerance in algae. CRISPR/Cas-9 has been successfully adapted for use in marine algae, including brown macroalgae, and can now be used to validate candidate knockout mutations (Nymark et al., 2016; Badis et al., 2021). However, its current regulation in farming is strict, limiting its widespread application in crop development.

## Conclusion

Giant kelp holds great ecological and economic significance, serving as a cornerstone species in marine ecosystems and supporting diverse marine life. However, it faces challenges including habitat degradation, climate change impacts, and a lack of the genetic resources required for effective restoration efforts and to maximize its farming potential. To help address this issue, we established a comprehensive database of naturally occurring mutations in a seedbank containing 559 individuals and explored the effects of these gene mutations. We annotated over 15.9 million mutations in 25,900 genes of the giant kelp genome. While the results can provide valuable insights, it is important to note the limitations of this dataset, as only 8,000 of the genes have functional annotation and are placed in a KOG pathway. This underscores the need for improved gene annotation methodologies for brown macroalgae like giant kelp. Recent genomic advances in brown macroalgae research have the potential to significantly improve predictions of mutation effects and enhance the use of natural knockouts.

In conclusion, establishing this comprehensive mutation database represents a significant milestone in conserving and sustainably managing giant kelp populations. By leveraging genetic insights, we now have a valuable resource to guide restoration efforts and identify preexisting gene knockouts for breeding programs aimed at cultivating resilient and productive kelp farms.

## Materials and methods

Details on sporophyte collection, gametophyte isolation, DNA extraction and sequencing, farm design, and phenotyping are described in Osborne et al (Osborne et al., 2023). Briefly, sequencing was performed on an Illumina S4 Novaseq platform (150 bp paired-end) at the BGI North American NGS lab, generating approximately 11.2 GB or 87 million reads per sample. Raw reads were then trimmed using fastp (version 0.20.1) (Chen et al., 2018). Trimmed reads were then aligned to the giant kelp reference genome using hisat2 v2.1 with standard parameters (Tajima, 1989). The genome-wide coverage per sample was approximately two to three times the expected depth (Kim et al., 2019; Diesel et al., 2023). Bam files had their duplicates marked using the GATK4 v4.1.2 command “MarkDuplicates”, and then multiple bam files for a single individual genotype were collapsed into a single bam file using Samtools (Li et al., 2009; Van der Auwera et al., 2013).

Genetic variants were called using the GATK4 v4.1.2 with a ploidy set to 1 (Van der Auwera et al., 2013). Individual GVCF files were then merged and converted into a raw VCF file containing variant information and used for downstream applications using GATK v4.1.2 (Van der Auwera et al., 2013). The raw nuclear VCF file containing 559 individuals, was then filtered for downstream genotype-phenotype modeling applications based on vcftools and GATK4 best practices (Van der Auwera et al., 2013; Danecek et al., 2021). Mutation classification was done with Snpeff (Cingolani et al., 2012). A nonstandard database was created using the giant kelps genome, and the General Feature Format (GFF) is publicly available from the JGI algal genome portal PhycoCosm (https://phycocosm.jgi.doe.gov/Macpyr2) (Grigoriev et al., 2021; Diesel et al., 2023). SnpEff was run with standard settings. Bcftools view -r was used to generate gene-specific vcf files (Danecek et al., 2021). For each gene, a vcf file was read into R using vcfR (Knaus and Grünwald, 2017), and hierfstat was used to calculate Fst, nucleotide diversity, and Tajima’s D (Goudet, 2005).

## Data Availability

The original contributions presented in this study are publicly available. This data can be found here: https://www.ncbi.nlm.nih.gov/sra/PRJNA1050779.
